# HSCT in SAVI: evidence for a curative hematopoietic approach and perspectives for gene therapy

**DOI:** 10.1016/j.omta.2026.201784

**Published:** 2026-06-30

**Authors:** Jade Cognard, Sébastien Viel, Alexandre Belot

**Affiliations:** 1 International Center of Research in Infectiology, Lyon University, INSERM U1111, CNRS UMR 5308, ENS, UCBL, Lyon, France; 2 National Referee Centre for Pediatric-Onset Rheumatic and Autoimmune Diseases (RAISE), Pediatric Nephrology, Rheumatology, Dermatology Unit, HFME, Hospices Civils de Lyon, 69677 Bron Cedex, France; 3 Plateforme ARTEMIS, Hôpital Edouard Herriot, Hospices Civils de Lyon, Lyon, France

## Main text

Since its first description in 2014 by Raphaela Goldbach-Mansky, stimulator of interferon gene (STING)-associated vasculopathy with onset in infancy (SAVI) has been reported in less than one hundred cases and more than thirty genotypes. SAVI is a rare and severe monogenic interferonopathy caused by autosomal dominant gain-of-function mutations in *STING1* (or *TMEM173*), encoding the STING protein.^1^ STING is a central adapter molecule of innate immunity, downstream cGAS, a cytosolic DNA sensor, and is ubiquitously expressed, with particularly prominent functional roles in immune and endothelial cells. Constitutive STING activation drives chronic type I interferon (IFN-I) production and persistent activation of inflammatory signaling pathways. Clinically, SAVI manifests as systemic inflammation, severe cutaneous vasculopathy with acral ulcerations, and progressive interstitial lung disease which can be fatal before adulthood.

In a recent issue of *Molecular Therapy Advances*, Yu, Song et al. reported the first successful allogeneic hematopoietic stem cell transplantation (HSCT) in a child with severe SAVI, caused by a heterozygous *de novo STING1* p.N154S mutation.^2^ Prior to transplantation, the patient had failed JAK inhibition including ruxolitinib, tofacitinib, and baricitinib and presented with progressive pulmonary fibrosis, persistent cutaneous ulceration, and marked depletion of CD8^+^ central memory T cell. Following myeloablative conditioning and infusion of peripheral blood stem cells from a fully HLA-matched sibling donor, full donor chimerism (>99%) was achieved. At 12 months post-HSCT, cutaneous vasculopathy had completely resolved, lung function showed marked recovery, and inflammatory markers normalized, without graft-versus-host disease (GVHD) or major infectious complications. CD8^+^ central memory T cells had reconstituted to normal levels and remained stable during cyclosporine tapering.

This case provides an important proof of concept that replacement of the hematopoietic compartment alone may be sufficient to induce durable remission in SAVI, with direct implications for the design of curative strategies, including allogenic HSCT and autologous gene therapy approaches. It also raises an important unresolved question about whether residual mutant STING in non-hematopoietic cells, particularly in the vascular endothelium, will ultimately limit long-term disease control.

The pulmonary outcome reported by Yu, Song et al. warrants particular attention. Lung disease is the principal determinant of morbidity and mortality in SAVI, and current medical treatments, including JAK inhibitors and IFNAR1 blockade with anifrolumab, consistently achieve only partial, and often transient, attenuation of pulmonary inflammation. Across published cases, lung transplantation has been associated with poor outcomes, including early graft failure and fatal disease progression, even when performed under JAK inhibitor therapy and standard immunosuppression.^3^^,^^4^^,^^5^ These cumulative observations strongly suggest that replacement of the pulmonary parenchyma alone is insufficient to interrupt the pathogenic process in SAVI. Mechanistically, this is readily explained: although the transplanted lung expresses wild-type STING in its stromal and endothelial compartments, it is exposed to the same pool of T cells and monocytes that drove inflammation in the native lung. In this context, achieving durable pulmonary remission likely requires correction of the aberrant hematopoietic source in addition to the replacement of the tissue target.^3^

The rationale for targeting the hematopoietic compartment in SAVI is further supported by preclinical studies in murine models, although no single model fully recapitulates the human disease. In STING gain-of-function models such as the N153S and V154M mice, lung inflammation can develop independently of IFN-I receptor signaling, arguing against a purely interferon-driven pathology.^6^ Importantly, pulmonary disease in these models critically depends on adaptive immunity: mice lacking T cells are largely protected and mutant hematopoietic cells are sufficient to transfer lung disease to irradiated wild-type recipients. These experiments collectively identify hematopoietic cells, particularly αβ T cells, as major drivers and amplifiers of pulmonary pathology.^7^ Although caution is required when extrapolating murine findings to humans, these data provide a compelling mechanistic framework supporting HSCT as a rational curative strategy in SAVI.

However, human observations raise important questions regarding the contribution of non-hematopoietic cells, particularly endothelial cells, to SAVI pathogenesis. Clinical heterogeneity among patients carrying different *STING1* variants suggests that endothelial pathology may, in some settings, be partially uncoupled from systemic interferon activation.^8^ Supporting this notion, conditional mouse models have demonstrated that endothelial-specific expression of STING gain-of-function mutations is sufficient to drive pulmonary immune-cell recruitment and bronchus-associated lymphoid tissue formation, even in the presence of a wild-type hematopoietic compartment.^9^ In parallel, *in vitro* and *in vivo* studies indicate that cytokines produced by mutant immune cells can further exacerbate endothelial activation and injury, establishing a feedforward inflammatory loop.^10^ Together, these observations support a two-tier pathogenic model in SAVI: endothelial STING activation may initiate local inflammation, while hematopoietic immune cells amplify and sustain the process to its full pathological expression.

Beyond clinical improvement, the detailed immune reconstitution data reported in this case provide valuable mechanistic insights. Following HSCT, immune recovery followed expected myeloablative kinetics, with normalization of B cell number and correction of prior humoral activation, reflected by normalization of immunoglobulin levels. T cell reconstitution was notable for recovery of effector CD8^+^ T cells consistent with post-transplant homeostatic proliferation. Clinically, immune reconstitution was paralleled by substantial functional gains, including catch-up growth, marked reduction in disease-related symptoms, and recovery of motor abilities that had been lost prior to transplantation. Together, these findings indicate that hematopoietic correction not only suppresses over inflammation but also restores immune and functional competence.

The success of allogenic HSCT in this case provides both therapeutic validation and a strong mechanistic rationale for the development of autologous gene therapy (GT) in SAVI and, more broadly, in monogenic interferonopathies. Autologous GT could overcome key limitations of the allogeneic transplantation, including GVHD, and the need for fully HLA-matched donors. They could also enable the use of non-genotoxic or reduced-intensity conditioning regimens, an important consideration for patients with advanced pulmonary disease. However, SAVI poses specific challenges for GT, as pathogenic mutations are dominant and heterozygous, necessitating correction of a substantial proportion of hematopoietic stem and progenitor cells to restore physiologic STING signaling. Recent preclinical work, currently available as a non-peer-reviewed preprint, had begun to address this challenge using genome editing approaches combining allele disruption and functional gene replacement in human CD34^+^ cells and patient derived induced pluripotent stem cells (iPSCs). This study suggests that approximately 90% allelic correction may be required to suppress spontaneous IFN-α production in the myeloid lineage, while preserving long-term repopulating capacity.^11^ Although these findings are promising, several key questions remain unresolved. Chief among them is whether the lymphoid compartment will be equally corrected and capable of generating a fully functional, mature T cell repertoire. Pharmacological STING inhibitors, which have shown partial normalization of constitutive signaling in cellular models, may complement genomic approaches, particularly for targeting residual non-hematopoietic STING activity that is not addressed by hematopoietic correction alone.^12^

**Figure 1:**
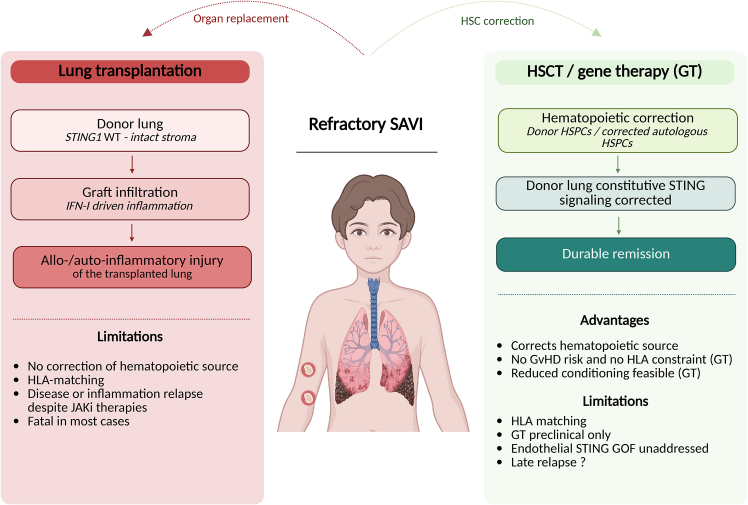
Therapeutic strategies in JAK inhibitor-refractory SAVI SAVI, STING-associated vasculopathy with onset in infancy; JAKi, Janus kinase inhibitor; IFN-I, type I interferon; HSCT, hematopoietic stem cell transplantation; HSPC, hematopoietic stem and progenitor cell; GT, gene therapy; GOF, gain of function; GvHD, graft-versus-host disease.

In summary, the case described by Yu, Song et al. provides the first in-human evidence that correction of the hematopoietic compartment can suppress SAVI-associated inflammation over a clinically meaningful period. While the 12-month remission observed is highly encouraging, it cannot yet be interpreted as definitive cure. Residual endothelial STING activation remains a plausible source of late relapse, and murine models cannot fully resolve this question due to fundamental species differences in pulmonary vascular biology. Long-term follow-up, including careful monitoring of pulmonary and vascular inflammation, will therefore be essential. Should durable disease control be confirmed, this case will establish the hematopoietic inflammatory drive as the rate-limiting component of SAVI pathology, and validate its correction, whether through allogeneic HSCT or autologous GT, as a viable path toward cure. If relapse occurs, combining hematopoietic correction with targeted inhibition of residual non-hematopoietic STING activity may represent a rational next step. This report thus provides a critical clinical foundation on which future therapeutic strategies for SAVI can now be built (Figure 1).

## Declaration of interests

A.B. is a co-author of Berrada KR et al., J Clin Immunol 2023, cited in this commentary. A.B. and S.V. are co-authors of Pavel-Dinu M, Viel S et al., Human Stem Cell Genome-Editing 2024, also cited in this commentary.

Acknowledgements

A.B. acknowledges funding from the Agence Nationale de la Recherche (ANR; grant numbers ANR-21-CE17-0064 [SOCSIMMUNITY] and ANR-21-RHUS-08 [COVIFERON]) and from Horizon Europe (grant number HORIZON-HLTH-2021-DISEASE-04 [UNDINE]).
